# Detecting bacterial adaptation within individual microbiomes

**DOI:** 10.1098/rstb.2021.0243

**Published:** 2022-10-10

**Authors:** Tami D. Lieberman

**Affiliations:** ^1^ Department of Civil and Environmental Engineering, Institute for Medical Engineering and Science,Massachusetts Institute of Technology, Cambridge, MA, USA; ^2^ Broad Institute, Cambridge, MA, USA; ^3^ Ragon Institute, Cambridge, MA, USA

**Keywords:** human microbiome, microbial evolution, parallel evolution, bacterial genomics, adaptation

## Abstract

The human microbiome harbours a large capacity for within-person adaptive mutations. Commensal bacterial strains can stably colonize a person for decades, and billions of mutations are generated daily within each person's microbiome. Adaptive mutations emerging during health might be driven by selective forces that vary across individuals, vary within an individual, or are completely novel to the human population. Mutations emerging within individual microbiomes might impact the immune system, the metabolism of nutrients or drugs, and the stability of the community to perturbations. Despite this potential, relatively little attention has been paid to the possibility of adaptive evolution within complex human-associated microbiomes. This review discusses the promise of studying within-microbiome adaptation, the conceptual and technical limitations that may have contributed to an underappreciation of adaptive *de novo* mutations occurring within microbiomes to date, and methods for detecting recent adaptive evolution.

This article is part of a discussion meeting issue ‘Genomic population structures of microbial pathogens’.

## 1. Introduction

While much attention has been paid to the ecology of microbiomes within individual people—which bacterial species reside within us during health [1,2], how these species interact with one another [3–5], and how they interact with the host [6,7]—relatively less attention has been paid to the evolution of single species and strains within these communities. Yet, there is a large potential for adaptive evolution within individual human microbiomes [8], enabled by population sizes greater than 10^12^ cells per person [9]. Mutations that confer antibiotic-resistance arise during infections and outcompete their ancestral genotype within days [10], and individual strains can persist and diversify within a person's microbiome for decades [11]. As such, individual strains have ample opportunity to explore potential adaptive mutations and change significantly during their residence within a host.

To date, studies of microbiome evolution have largely focused on relatively long timescales—how our microbiomes have changed as mammals [12], primates [13], or human populations diversified [14]. These studies have characterized how host evolution has selected for different bacterial communities, defined at the species or higher taxonomic level using 16S rRNA sequencing [15,16], but less attention has been paid to how those species and strains themselves might be changing. Even when studies focus on genomic changes within a species, these have mostly focused on timescales considerably longer than a single human lifetime, such as the absence of motility operons within a subspecies of *Eubacterium rectale* that is largely restricted to Europe [17].

Yet, tracking bacterial adaptation at short timescales, particularly within individual human microbiomes, can yield both practical and conceptual insights. From an applied perspective, identifying genes and genomic loci undergoing adaptive evolution in real time provides insight into the selective pressure experienced by microbes *in vivo* [18,19] and therefore aids in the design of rational probiotic therapies. Identifying genetic variants critical to bacterial survival in human microbiomes can be difficult using *in vitro* and laboratory animal models. Bacterial gene expression and survival strategies are highly context-dependent, and it is not uncommon for small shifts in the environment, such as inclusion of a community member, to alter the degree to which a genetic mutation is advantageous or deleterious in a laboratory setting. As an extreme example, studies of how bacteria adapt to survive high levels of an antibiotic must be carefully designed to avoid selecting for mutations that enhance adhesion to the walls of a test tube [20]. Organs-on-chips [21] and mice with humanized microbiota [22], while powerful, do not mimic *in vivo* conditions well enough to avoid system-dependent selective pressures [23]. By contrast, rigorous genomic signatures of in-host adaptation can conclusively indicate the presence of selection [8,19]. The identification of such adaptive changes provides a starting point for hypothesis-generation regarding selective forces, follow-up investigations using experimental, computational and other approaches [24], and engineering of probiotic therapies. For example, a study of the commensal *Bacteroides fragilis* in healthy subjects revealed single nucleotide changes in importers of complex polysaccharides that provide strong selective advantages; these changes might reflect a metabolic challenge or, alternatively, pressure from phage that use these proteins as gateways into the cell [25]. Accordingly, the rational design of *Ba. fragilis* probiotics should include selection of strains with the ability to thrive in the presence of these selective pressures.

On a conceptual level, understanding the extent to which microbes evolve within individual microbiomes is critical to understanding microbe–disease association and building predictive models of microbiome assembly. Adaptive mutations emerging within microbiomes might impact how a bacterium interacts with the immune system, the metabolism of nutrients or drugs, or the stability of the community to perturbations. If adaptive mutations are common within microbiomes, understanding the impact of a particular species or strain on host health or community composition might require full-genome resolution. In support of this idea, a recent study identified that loss-of-function mutations in a particular gene of *Staphylococcus aureus* is associated with the inflammatory skin disease atopic dermatitis [26], even though no study has found strains or phylogroups of this species associated with disease. Moreover, adaptive mutations might change the way we think about microbiome stability and resistance to perturbation [5,27]. Adaptation within individual microbiomes might be driven by selective forces that are new to industrialized societies, vary across individuals, or vary within individual people. If person-specific selective forces are common, it is possible that the bacteria within each person's microbiome may have already adapted, at least in part, to unique selective forces in their community [28]; this early adaptation may explain the resistance of established microbiomes to invaders [2,11].

Lastly, tracking evolution at short timescales is critical for understanding the mechanisms and selection modes (e.g. purifying versus positive) responsible for genetic variation within the microbiome [29]. Rates of genetic change in bacteria, as well as signals for adaptive evolution, are highly dependent upon the timescale separating the compared genomes [30–33]—estimates are faster [32] and more biased towards amino acid changes [33] when comparing closely related organisms than more distant ones. Timescale dependence makes it difficult to predict the relative likelihoods of mutations versus recombination, adaptive versus deleterious alleles and ecological versus evolutionary responses to perturbations within individual microbiomes [31,34]. Tracking evolution within individual microbiomes provides an opportunity to avoid this timescale dependence and quantify such real-time evolution in natural settings. Understanding the forces that create genetic variation within microbiomes will inform the degree to which such variation should be considered when modelling microbiome ecology or predicting the impact of microbiome-based interventions.

It may seem surprising that, despite this potential, the first studies of within-person bacterial evolution in the microbiome at a genomic scale have only emerged within the past five years [8,35–38]. Within-person adaptation is probably underappreciated because of both theoretical misconceptions and technical limitations of the most popular approaches in the microbiome field. This review aims to reveal and dispel these misconceptions and describe one powerful roadmap for detecting within-person adaptation. Examples from both commensals and infectious disease will be used to build intuition, with a particular emphasis on chronic opportunistic infections of the cystic fibrosis (CF) lung, as the field of within-host infectious disease evolution is more established. No attempt will be made to comprehensively review all studies of within-host bacterial evolution; recent reviews focusing on bacterial pathogen evolution provide an excellent summary of within-host evolutionary trends, many of which also apply to commensal microbes [39–41]. Instead, this piece focuses on demystifying the processes and theoretical considerations for detecting within-microbiome adaptation, with the aim to accelerate work in this area.

## 2. Rates of evolutionary and ecological changes in microbiomes

Human microbiomes can be considered stable or dynamic, depending on the timescale and genomic resolution considered [2,11,42]. Each person has a unique set of microbial species across their total microbiome, though the functional capacity of microbiomes varies less from one person to another than the specific membership [1]. The species [2,42] and strain [11,43] composition of a person's gut microbiome is remarkably stable on the timescale of a year, though the relative abundances of these organisms can fluctuate substantially on daily timescales [42,44].

The stability of human microbiomes at the level of membership is thought to emerge in part from priority effects [45,46]—the ability of early colonizers to alter community formation and exclude later migrants [47]. Accordingly, perturbations that lower bacterial abundance, including antibiotic treatment, are associated with an influx of newly colonizing strains in the gut [5,48]. Adult siblings share few strains in common [11], suggesting that such disturbances may be relatively common, although the likelihood that these sibling microbiomes were seeded by different organisms in early life has not yet been resolved.

**Figure 1.**
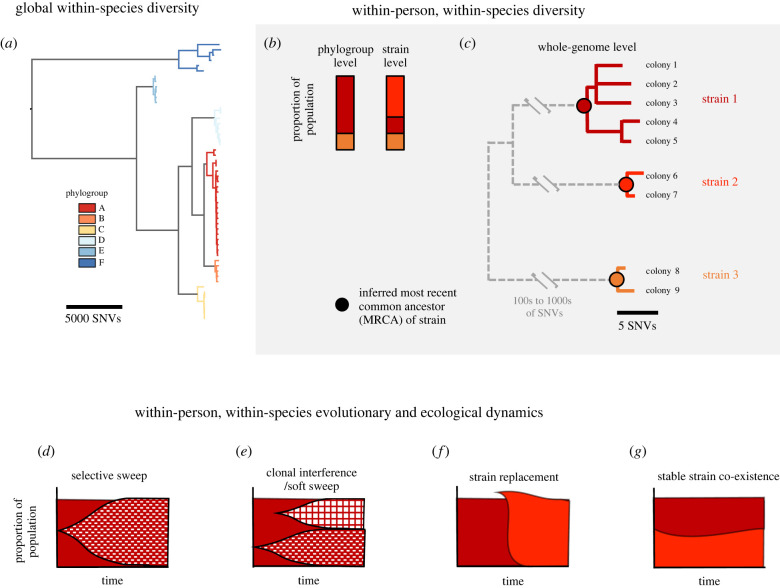
Subspecies classification and dynamics. (*a*) Within-species diversity of bacteria can be described as a phylogenetic tree and can be defined at multiple phylogenetic levels. Here, phylogroup is used to mean groupings of isolates separated by thousands of single nucleotide variants (SNVs) across the whole genome. (*b*,*c*) Within-person, within-species diversity can be described at multiple levels of resolution; the example has three strains of the studied species, two of which are from the same phylogroup and indicated in different shades of red. (*c*) Strains are defined here as groups of colonies separated by less than 100 SNVs across the genome; this cut-off is designed to group colonies that could have diversified on the host from a single cell; the exact cut-off used depends on the molecular clock rate of that species. (*d*,*e*) illustrate two types of subspecies evolutionary dynamics at the substrain level, while (*f*,*g*) illustrate ecological dynamics at the strain level.

There are many levels of resolution at which one can define a strain or other subspecies grouping. In this review, the primary unit of organization is a ‘strain’, defined as a monophyletic grouping of very closely related genotypes inferred to have a single-cell common ancestor within approximately the past 100 years (figure 1*c*; this has also been termed a ‘cloud’ [49]). The mutational distance cut-off to define a strain depends on the molecular clock of the studied organism, but it is often on the order of 100 mutations across the whole genome, as molecular clock rates in commensal and pathogenic bacteria do not vary that much—from 0.5 to 8 mutations genome^−1^ yr^−1^, with the slow-growing *Mycobacterium tuberculosis* on the low end and *S. aureus* on the high end [41]. Exceptions must be made for hypermutators that accumulate mutations at higher rates owing to defects in DNA repair [50], and regions of the genome undergoing recombination or horizontal gene transfer must be removed before defining molecular clock rates or strain boundaries.

Given this relatively slow molecular clock range, it may seem surprising that beneficial mutations can be supplied at high rates within individual microbiomes. However, mutation supply and the molecular clock are distinct concepts. Each bacterial cell residing within a microbiome can supply random mutations created during DNA replication each bacterial generation; only the surviving mutations along a single line of descent are measured in calculation of molecular clocks.

**Figure 2.**
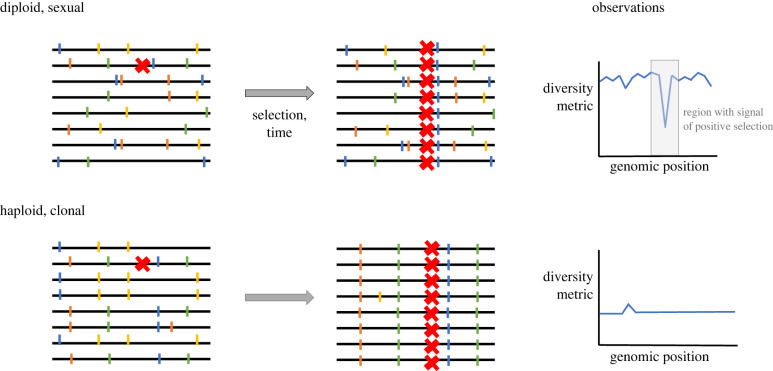
Recombination enables strong signatures for positive selection in recombining organisms but not in primarily asexual (clonal) populations. Recombination breaks up linkage between alleles in organisms that undergo frequent recombination. Red crosses indicate adaptive mutation. Other neutral alleles are represented by coloured vertical bars. When a selection event occurs, the adaptive mutation is driven to high frequency. In a highly recombining organism, this only drags very close alleles to high frequency, and thus a deviation from genome-wide metrics of diversity can be observed. However, in a primarily clonal population the population-wide sweep of an adaptive allele causes all diversity to be lost, at least until new mutations emerge. This process is known as genomic draft or hitchhiking.

During the years and decades that a strain stably persists within a microbiome, its member organisms thus acquire *de novo* mutations that create new genotypes and ‘substrains’ (figure 1*c*). Strains with substantial substrain structure have been found in a variety of infections [49,51–54], gut commensals [8,37,38], and skin commensals [36,55,56]. The amount of diversity within a strain can be used to infer lower bounds on its duration of residence in a microbiome [8,52]. This approach can overestimate the duration of colonization if within-person populations are founded by closely related cells, rather than from a single-cell bottleneck. On the other hand, residence time can be overestimated following an adaptive sweep (figure 1*d*) or neutral population bottleneck. Notably, not all adaptive mutants that rise in frequency lead to population-wide sweeps; it is frequent for competition between adaptive alleles on different genomic backgrounds (figure 2), termed clonal interference [57] or soft sweeps [58,59], to lead to coexistence of substrains [8,52,60].

The relative infrequency of immigrating strains and competitive between-strain dynamics (figure 1*f*) observed to date in human microbiomes [11] suggests that opportunity remains for *de novo* mutations to contribute to competitive dynamics (e.g. response of community to a new phage). Adaptive substrain dynamics, including adaptive sweeps, diversification, and clonal interference, have been shown to be common for *Ba. fragilis* [8] and other species [11,37] in the human gut, as well as *S. aureus* on the skin of children with atopic dermatitis [61]. While detection of some of these dynamics is possible with metagenomics [11], approaches that yield high-quality single-cell genomes are required to build phylogenetic trees and get a full understanding of substrain structure. Current single-cell genomic techniques are too error prone and sparse in their coverage of the genome. Instead, culture-based approaches that use the fact that colonies are typically founded by single cells, and that minimize growth in the laboratories, have proved successful for reconstructing within-person evolution [8,36,38,56,61,62]. Using such culture-based approaches, adaptive substrain dynamics have been uncovered even in the presence of multiple-strain colonization in humans and mice [37,60].

## 3. Asexual within-host populations evolve differently to classical eukaryotic populations

Fundamental differences between evolution of haploid organisms, with primarily asexual reproduction, and that of classic, sexually recombing diploid organisms, has led some to underestimate the potential of adaptive evolution during colonization of a single host. Well-established models of adaptation in classical eukaryotes [63] create a strong intuition: the potential of a population to adapt increases with the amount of nucleotide diversity present within that population (π). Adaptive potential can be predicted from nucleotide diversity in classic diploid populations, which undergo one recombination event per chromosome per generation, for two reasons. First, a higher nucleotide diversity enables rearrangement of existing alleles into more novel genotypes with the potential for enhanced fitness. Second, high nucleotide diversity in a recombining population is considered to reflect a large population size and therefore predict a high rate of emergence of new, potentially adaptive, *de novo* mutations in the population. It is no wonder, then, that extremely low levels of within-person nucleotide diversity [64] are sometimes treated as severe limitations on adaptive potential.

Yet, the shuffling of alleles via recombination is likely to be rare for most within-person bacterial populations. As bacteria can only undergo homologous recombination with closely related individuals, usually of the same species [65], the low number of coexisting strains of any given species within a given microbiome [11,37] limits the supply of alternative alleles for any given gene (horizontal gene transfer is a distinct process from recombination, which brings in new alleles of existing genes). Thus, even if a bacterial strain is naturally competent [66] or has the capacity for phage-mediated gene transfer [67,68], the potential for bringing in new alleles via recombination is low; most recombination events will occur within members of the same strain. This low level of recombination means that measures of nucleotide diversity and adaptive potential are decoupled; while a two-strain population has access to a larger genotypic space than a single-strain population, this increase is not proportional to the corresponding change in π.

This genome-wide linkage limits the number of unique genotypes that can be found within each host [69]. When a strongly adaptive mutation arises in the population, it is unlikely to recombine and be found on a genomic background other than on the one on which it arose; instead, it is likely to drag all mutations on its genomic background—including neutral ones—to high frequency in a process known as genetic draft [70,71] (figure 2). Genetic draft, also termed hitchhiking, is not unique to microbial systems [72], but its impact here is large owing to genome-wide linkage. In this way, an adaptive sweep can purge all existing diversity and produce a population founded from a recent single cell. Therefore, positive selection and genetic draft keep *π* low, despite years of colonization in a host, during which diversity could theoretically accumulate.

Instead, the greatest predictor of adaptive potential in within-host populations is likely to be the census population size—how many bacteria cells of a species are reproducing at a given time. This is because the supply of *de novo* mutations scales linearly with the number of cells in a population. For many bacterial species, the number of cells within an individual human host, and therefore the supply of mutations, is enormous. A minor species (0.1% abundance) in a person's gut can easily be comprised 10^10^ cells [9]. Given a conservative bacterial mutation rate of 10^−10^ mutations per cell division [73], such a population can explore every single point mutation across the genome each generation (probably days) [8].

## 4. Challenges in detecting recent within-host bacterial adaptation

Adaptation in asexual populations is also probably underestimated owing to technical challenges in distinguishing adaptive from neutral variants. A wide variety of statistical tests have been developed to identify genomic regions under recent pressure to change in classically sexual populations [24,74]. For the most part, these tests compare diversity metrics within (such as *π*) or across populations (such as *F*_ST_) to what would be expected under a neutral model with no adaptation. A widely acknowledged limitation of such tests for adaptation is that confounders, including spatial structure and changes in population size, can dramatically skew these metrics [24]. Therefore, it is standard practice to compare values of diversity metrics at a candidate locus to those obtained in the rest of the genome. This approach works because recombination in sexual organisms enables genomic regions to have independent diversity metrics. In primarily asexual organisms, however, loss of diversity at one site often can cause loss of diversity throughout the genome (figure 2). Without an internal control for these diversity metrics, it can be difficult to confirm a particular locus has recently undergone adaptive evolution.

The other major category of tools for identification of adaptive evolution relies on the fraction of mutations that are nonsynonymous (N), or amino acid changing, rather than synonymous (S), or amino acid preserving. Because of redundancy of the genetic code, only approximately 75% of mutations in coding regions are expected to change the amino acid code (the exact percentage depends on the codon usage and spectrum of mutations of a given organism). Therefore, adaptive evolution can be detected as a statistically significant increase in the percentage of mutations that are non-synonymous relative to a calculated expectation (e.g. dN/dS) [75], to an expected value from between-species substitutions (e.g. McDonald-Kreitman) [76–78], or to observed values from other areas of the genome [79]. Critically, N versus S analyses must be done on a small segment of genomes, such as genes, protein domains, or even single nucleotides [78]. As most sites in a genome are under pressure to retain their amino acid sequence (purifying selection), signals of adaptive evolution can disappear when N versus S analyses are performed genome-wide [19].

However, a major challenge of N versus S approaches is the number of mutations needed to observe a statistically significant enrichment in the percent of mutations that are N (p_N_). This difficulty arises because p_N_ is so high under neutral expectation. For example, to reject a null model of 75% N, at least 11 N mutations must be observed—under such a null model, 10 of 10 mutations being non-synonymous is not statistically significant to a *p* = 0.05 significance threshold (one-sided binomial test). This calculation only reflects a lower bound on the number of mutations, as it assumes no neutrally drafted or adaptive synonymous mutations in the gene of interest. Moreover, this simple calculation omits the necessary step of multiple-hypothesis correction for genome-wide scans. Therefore, using N/S ratios to detect genes undergoing adaptive evolution often requires dozens of observed mutations per gene, or more. When assessing within-person adaptation, it is rare to observe such a high number of mutations in a single gene. Typical datasets, even across cohorts of over a dozen individuals, include only hundreds or low thousands of *de novo* mutations across the genome [8,62,80]. Therefore, methods relying on p_N_ or dN/dS are better suited to the larger number of mutations generated on longer timescales or aggregating over sets of genes.

## 5. Parallel evolution as a powerful tool for detecting recent within-host adaptation

One approach with demonstrated success in detecting bacterial genes under adaptive evolution within individual hosts is scanning for parallel evolution (PE)—the emergence of similar mutations on distinct genomic backgrounds. Scans for PE start by counting the number of independent mutation events that occurred in a gene. This step requires phylogenetic analysis and is more complex than just identifying variable sites. Processes like recombination or repair of double-stranded DNA breaks can create multiple variant sites emerging from a single event [81,82]. On the other hand, a single variable site could emerge from two identical, parallel, mutations occurring in different genomic backgrounds [19,83]. Next, the number of mutations is compared to a neutral model in which mutations are randomly scattered throughout the genome [8,56]. While multiple-hypothesis correction remains an issue, approaches that use false-discovery rates (FDR), including simulations, have shown success in detecting meaningful signals of PE [8,52,84].

PE can only weakly support adaptive evolution—if considered in isolation. Signatures of PE could emerge if regions of the genome have intrinsically higher mutation rates. To confirm adaptive evolution, dN/dS or another secondary test can be applied to the set of genes with a signal of PE [8,19]. As tests such as dN/dS are difficult to interpret with small numbers of observed mutations, grouping all mutations within a set of genes under PE can provide sufficient signal. This two-step process is statistically valid because the number of mutations in a given gene and the proportion of those mutations which are N are independent. Thus, a set of genes with significant signals for adaptation using both approaches can confidently indicate the presence of adaptive evolution.

While the use of an FDR rather than strict hypothesis correction means that not all identified genes are truly under pressure to change *in vivo*, inspection of the function of genes can support the conclusion of adaptive evolution. For example, in a study of *B. fragilis* evolution in the gut of healthy people, 6 of 16 genes inferred to be under PE were all in the SusC/D family of complex polysaccharide importers (a significant enrichment relative to the per cent of genes with this annotation) [8], providing confidence that genes in this family are under pressure to change within individual healthy gut microbiomes. However, relying on gene function annotations currently remains challenging owing to the large number of unannotated or loosely annotated microbial genes; literature searches can often reveal functional roles not captured by ontologies [85]. An alternative signal that can provide confidence in the adaptive nature of observed mutations is the location of mutations within a given gene. For example, in a study of *Burkholderia dolosa* evolution during long-term infection of patients with CF, 12 of 17 mutations within the gene *fixL* fall within two small domains of this oxygen-dependent response regulator [19]. The clustering of these mutations, and the fact that none of the mutations created premature stop codons, provided confidence that *B. dolosa* is under pressure to tweak, but not break, this response regulator *in vivo* [86].

While searches for parallel adaptive evolution have traditionally considered single-nucleotide variants, larger genomic changes can also drive adaptive evolution, e.g. the gain of a mobile element or deletion of a gene [87,88]. However, the adaptive nature of a given mobile element change can be more difficult to identify from a genome-wide analysis because neutral expectations of the number of changes are difficult to model (as such changes are not clocklike) and there is no accepted independent test for selection that is analogous to dN/dS. Instead, focused investigation of candidate regions has proven successful in confirming the adaptive nature of horizontal gene transfer events [87,89].

## 6. The power of within-person parallel evolution

PE can be considered at genomic scales (e.g. gene versus pathway) and geographical scales (e.g. between people versus intra-person). This section discusses the data needed to detect within-host mutations and then explains the surprising success of within-individual PE relative to across-individual PE.

To identify variants that emerged during colonization, it is important to compare many genomes from each person and strain studied. These isolates can be collected over time, collected from the extant coexisting diversity within a host at a single time point, or both. Typical studies profiling cross-sectional diversity collect 10–100 colonies from each person at a given timepoint [8,26,36,62], with the ideal number to capture variation depending on the species and sampling site.

**Figure 3.**
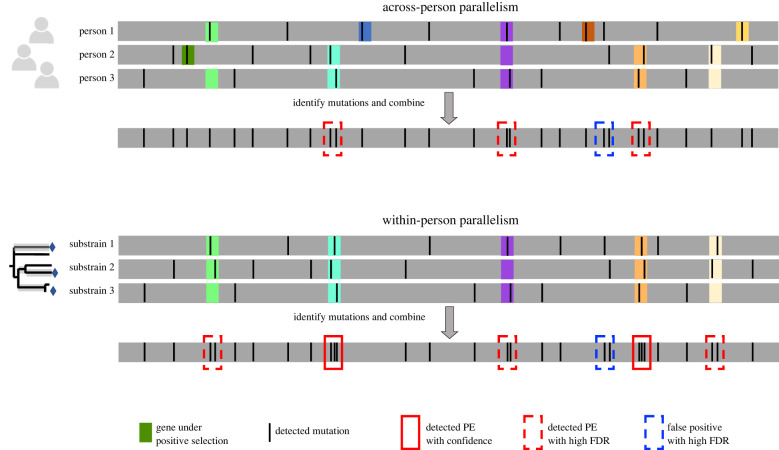
Across-person analyses lose statistical power in the presence of person-specific adaptation. Each line represents a bacterial genome from a single colony isolate. Shaded areas represent genes under positive selection shaded. In the top example, *de novo* mutations are identified separately in three subjects. As the genes under selection are not known ahead of time, a search for parallel evolution identifies only four genes mutated twice, and no genes mutated more times than that. One of these genes is a false positive (a gene not under directional selection). By contrast, when a single person is sampled more exhaustively such that more mutations which occurred within this subject are detected, two genes are confidently identified as under selection (three mutations each) owing to the uniformity of selective pressures across the sampled substrains. These examples illustrate how the presence of non-adaptive mutations creates statistical noise and a high bar for the confident detection of selection. Therefore, approaches which minimize the variation in selective forces between considered replicates have the most statistical power. Similar signal-to-noise issues can arise when comparing mutations at different stages of colonization or considering genes at the pathway level.

It is not sufficient to compare just one isolate each from many individuals. Thousands of mutations typically separate isolates from different individuals, most of which occurred in the distant past. Studies comparing bacterial genomes separated by various timescales have shown that adaptive signals strongly depend on the timescale studied [8,11,31,33,90]. One possible contributor to this timescale dependence is that proportionally more neutral mutations reach detection on longer timescales, via both random between-host transmission bottlenecks and hitchhiking [71] on adaptive sweeps—which can overwhelm the few loci undergoing adaptive evolution. In addition, selective forces can vary over time, creating variation that obfuscates signatures of parallelism and adaptive evolution (figure 3) [91,92].

Once a list of variants occurring within each individual has been obtained, genomic scans for PE can be performed on the list of all mutations (across-individual PE) or within individual subjects (within-individual PE). The only difference is the null model used: either a single model in which all mutations found are scattered randomly across the genome or a separate null model for each subject. Surprisingly, higher enrichments for non-synonymous mutations have been found when performing analyses at the within-individual level as compared with the across-individual level. In the previously mentioned study of *B. fragilis* evolution in 12 healthy human gut microbiomes, 16 genes were identified to be mutated at least twice within one or more subjects, compared to just five expected under a neutral model (a density threshold was used to exclude excessively long genes) [8]. When the same set of mutations was considered for across-individual PE, eight genes fell below the threshold for PE while only two additional genes were implicated in PE. Interestingly, these two new genes found in the across-person analysis had a lower p_N_ than expected from a neutral model (though not significantly). Studies of other bacterial species have also found similar strength of within-individual PE scans [53,93], with less sensitivity at the across-host level [8,26,94].

Why should an enrichment of mutations be observed from within-person analyses but not when the same data is aggregated across subjects? One possibility may be that selective forces are person specific. In the study of *B. fragilis,* the SusC-family gene *ccfC* was mutated three times in subject 1 and no times in other subjects. This raises the possibility that *ccfC* was under pressure to change owing to the unique set of selective forces in subject 1—imposed by their genetics, their behaviour, their immune system, or their microbiome.

Alternatively, this apparent ‘person-specificity’ could arise from differences in genomic background of each person's bacterial strain. Genomic background can diversify the response to selective pressure in both obvious and subtle ways. Most simply, divergent strains of a species with different gene content might overcome the same challenge using different mechanisms [95]. More subtly, genetic interactions (epistasis) between small mutations can alter the course of evolution. An interesting example comes from a study of *Pseudomonas aeruginosa* evolution during long-term infection of 34 patients with CF [80]. Mutations in the transcriptional regulator gene *algU* were observed under PE across subjects in that study. Yet, these *algU* mutations only occurred on genomic backgrounds that had already obtained a mutation in the transcriptional regulator gene *mucA.* Even subtler yet, *de novo* mutations that have already swept a within-individual population prior to sampling might have already alleviated a selective pressure [26].

Because of the importance of ever-changing genomic backgrounds, it can be easiest to detect within-individual PE when multiple solutions to the same challenge are coexisting on the same genomic background—before any of these parallel adaptive alleles has ‘won out’. Thus, coexisting adaptive alleles are easiest to observe via the sequencing of many cross-sectional isolates from a single timepoint [52,96]. In this light, cross-sectional sampling can be more sensitive for detecting loci under adaptation than longitudinal sampling [8,52]. On the other hand, longitudinal studies can be used to estimate the selective advantage provided by a mutation [8,11], understand dynamics [8,10,59], and even observe reversions [19,80,92].

Identifying coexisting genotypes with alternative adaptive mutations is not possible for all species and environments. Not all bacterial populations enable competing adaptive mutations to persist for long enough to be detected, and bacterial diversity can be lost rapidly if population sizes are small [38] or a rare adaptive mutation is substantially more advantageous than other available mutations [97]. In these cases, detection of within-person PE may be challenging, and screens for across-individual PE (of within-person mutations) will be relatively more powerful. Across-individual PE is particularly useful when selective pressures are strong and uniform; this approach has been one of the primary signals for identifying mutations which confer antibiotic resistance *in vivo* [10,98,99].

## 7. What is the right genomic unit for detecting adaptation?

At the genomic level, scans for PE can use nucleotides, codons, genes, operons, or pathways as the operative unit. In theory, larger genomic units enable more potential for PE signals to reach genome-wide significance. On the other hand, as genomic unit size increases, the chance also increases that the unit under consideration contains a mix of regions under directional selection (adaptive) and regions under purifying selection, thus erasing any signal [75]. An extreme example of this balancing effect comes from the previously mentioned study of *B. dolosa* evolution in infections of CF patients. In that study, genome-wide estimates of dN/dS were consistent with a neutral model even though a third of the intragenic mutations were concentrated in only 17 genes with highly elevated dN/dS. Signatures of purifying selection elsewhere in the genome neutralized the adaptive signal when averaged together [19]. Similarly, screens at the operon level only rarely detect cases of PE not detected at the gene level [52]. Yet, some new genes can be discovered at genomic units larger than the gene level, and it is relatively straightforward to test multiple genomic levels.

While, in theory, pathway-level screens for PE would remove some concerns regarding genome-specific solutions by categorizing mutations in broad functional groups, pathway-level annotations are not well-developed in microbial genomes. A large fraction of microbial genes remain hypothetical or unannotated, even in the most well-studied organisms. Even with pathway designations available, they are often rather coarse (e.g. transcriptional regulator instead of oxygen-dependent gene regulation). As a consequence, pathway sets in microbial genomes often contain very large sets of diverse genes, making it difficult to identify statistically significant signals.

For all these reasons, PE is easiest to detect using small genomic units and when positive selection is likely to be uniform across studied genotypes. In practice, this is often the within-gene, within-person level—particularly when many genomes or colonies from the same time point can be sampled.

## 8. Conclusion

The future of the field of within-microbiome evolution is bright, with many questions left to address and many potential implications for understanding and manipulating microbiomes. In the coming years, studies across a wide variety of species inhabiting human microbiomes will reveal the degree to which adaptation drives within-person commensal evolution and dynamics, as well as the relevance of bacterial adaptation to health, interspecies interactions, and community stability.

On a theoretical level, studies of within-host selection will be critical to understanding evolution of microbiomes across longer timescales. It has long been appreciated that the ability of a bacterial mutant to survive and compete within a local environment (e.g. a single human microbiome) may not predict its fitness across larger ranges of environmental variation [100,101]. The degree to which within-person adaptation is person-specific [8] or short-sighted [92] remains to be determined. Regardless, much of commensal competition, and thus adaptation, operates at the level of within-host communities owing to limited between-host migrations [11]. As such, understanding within-host adaptive events will be critical to modelling and predicting the long-term success of bacterial strains [34].

Should *in vivo* adaptations be predictable within or across individuals, we may one day be able to predict mutations conferring immune escape, antibiotic resistance, or disease before they happen. Should adaptive mutations be predictable within an individual, but distinct across individuals, this will suggest a large need for personalization when engineering microbiomes.

Those species which have the most capacity to change *in vivo* may be drivers of microbiome dynamics, colonization resistance, or disease pathologies. Interestingly, not all studies of within-person microbiome evolution to date have observed strong signals of adaptive evolution*.* Studies of *S. aureus* evolution during asymptomatic nasal carriage in 13 subjects [62], *Escherichia coli* in the gut of one healthy subject [38], and *Cutibacterium acnes* on normal facial skin of 16 subjects [56] have revealed only signals of purifying selection. While these organisms have relatively lower population sizes than *Bacteroides* [8] and pathogens of the CF lung [19,80], they are still thought to have population sizes greater than the bacterial per-base-pair mutation rate. For *C. acnes,* it has been speculated that adaptive potential is limited by the highly structured environment in which it resides, including genotype-agnostic single-cell bottlenecks within individual pores [56]. However, the roles of predation by phage, environmental variability and molecular mechanisms of non-genetic adaptation may also play a role in the balance of adaptive and neutral evolution. Understanding the factors that determine adaptive potential will focus efforts seeking to link in-person mutations to health and disease.

## Data Availability

This article has no additional data.
